# Possible involvement of viral infection in the pathogenesis of myasthenia gravis

**DOI:** 10.1007/s13365-026-01334-6

**Published:** 2026-08-26

**Authors:** Maria Dolci, Lucia Signorini, Federica Perego, Sara Passerini, Chiara Giannasi, Francesca Camurri, Alessia Berni, Sara Messina, Valeria Pietropaolo, Alessandro Baj, Paola Cavalcante, Serena Delbue

**Affiliations:** 1 Department of Biomedical, Surgical and Dental Sciences, University of Milano, Milan, Italy; 2 Department of Public Health and Infectious Diseases, “Sapienza” University of Rome, Rome, Italy; 3 Laboratory of Biotechnological Applications, IRCCS Ospedale Galeazzi–Sant’Ambrogio, Milan, Italy; 4 Neurology 4–Neuroimmunology and Neuromuscular Diseases, Fondazione IRCCS Istituto Neurologico Carlo Besta, Milan, Italy

**Keywords:** Myasthenia gravis, Viruses, Autoimmunity, Thymus

## Abstract

Myasthenia gravis (MG) is a chronic autoimmune disorder that involves the targeting of neuromuscular junctions and is primarily driven by autoantibodies against the acetylcholine receptor (AChR). While genetic susceptibility is a factor, environmental triggers, particularly viral infections, are being increasingly investigated as potential contributors to MG pathogenesis. This review examines the complex interplay between viral pathogens and MG, highlighting mechanisms such as molecular mimicry, bystander activation, and the aberrant stimulation of innate immune pathways via Toll-like receptors (TLRs). Key DNA viruses, including Epstein‒Barr virus (EBV) and parvovirus B19, have been detected within hyperplastic thymic tissues and thymomas, suggesting that they may sustain local autoreactive B-cell activation. Furthermore, recent clinical data emphasize the role of RNA viruses, most notably SARS-CoV-2, as significant triggers of new-onset MG and myasthenic crises. Despite substantial evidence linking viral infections to MG, further mechanistic research and large-scale observational studies are needed to definitively establish causality and refine therapeutic strategies targeting these virus-induced immune responses.

## Introduction

### Myasthenia: Disease and treatment

Myasthenia gravis (MG) is an autoimmune disorder of the neuromuscular junction (NMJ) characterized by fluctuating skeletal muscle weakness and fatigability caused by an autoantibody-mediated impairment of synaptic transmission (Kaminski et al. 2024). The disease is most strongly associated with autoantibodies against the acetylcholine receptor (AChR), which occurs in approximately 85% of patients with generalized symptoms (Lazaridis and Tzartos 2020). Less frequently, patients harbor autoantibodies against muscle-specific kinase (MuSK) or lipoprotein receptor 4 (LRP4), or they are seronegative for known autoantigens, suggesting the involvement of low-affinity antibodies, unknown antigenic targets, or antibody-independent immune mechanisms (Lazaridis and Tzartos 2020).

In AChR-MG, pathogenic antibodies belong predominantly to the IgG1 and IgG3 subclasses, leading to complement activation, membrane attack complex (MAC) formation, and structural damage to the postsynaptic membrane, highlighting the relevance of complement-mediated mechanisms in disease pathogenesis (Tüzün and Christadoss 2013). However, antigenic modulation with AChR endocytosis and the functional blockade of AChRs are additional autoantibody-mediated processes that lead to NMJ dysfunction (Khani-Habibabadi et al. 2025).

MG is a clinically and immunologically heterogeneous disease that can be classified into distinct subgroups according to the presence of pathogenic autoantibodies, clinical presentation, age at onset, and involvement of the thymus in its pathogenesis (Mantegazza and Cavalcante 2019). MG typically presents with ocular symptoms at onset (ocular MG) but later progresses to a generalized form (generalized MG) involving the bulbar, limb, and respiratory muscles (Mantegazza and Cavalcante 2019). Additionally, it is often categorized into early-onset (EOMG, < 50 years) and late-onset (LOMG, > 50 years) types, the immunopathological features of which differ (Gilhus and Breiner 2025). Thymic histopathology is a hallmark of AChR-MG. Thymic hyperplasia with ectopic germinal centers (GCs) is frequently observed in AChR-EOMG patients, whereas thymoma is associated with both EOMG and LOMG (Cavalcante et al. 2013). Chronic inflammation and Toll-like receptor (TLR)-mediated innate immune activation have been demonstrated to trigger and sustain anti-AChR autoimmunity in the follicular hyperplastic thymus of patients with MG through the local production of proinflammatory cytokines and aberrant antigen presentation accompanied by T- and B-cell dysregulation (Cavalcante et al. 2013; Robinet et al. 2017; Dragin and Le Panse 2025). In this context, an impairment of regulatory T cells (Tregs) can contribute to a global loss of immune regulation (Alahgholi-Hajibehzad et al. 2015). In both thymic hyperplasia and thymoma-associated MG, defects in T-cell selection and impaired Treg suppressive function have been observed and likely promote the expansion of autoreactive T and B cells that ultimately drive autoantibody production (Gradolatto et al. 2014; Chen et al. 2021).

The therapeutic management of MG follows a stepwise algorithm tailored to disease severity, autoantibody status, and the patient’s clinical features. First-line symptomatic treatment includes acetylcholinesterase inhibitors, while long-term disease control relies on immunosuppressive (IS) therapies, such as corticosteroids (e.g., prednisone) and nonsteroidal IS drugs (e.g., azathioprine) (Mantegazza and Antozzi 2020). Thymectomy is a therapeutic option for selected patients with AChR-positive generalized MG, even in the absence of thymoma, because of its demonstrated long-term clinical benefit compared with treatment with prednisone alone (Wolfe et al. 2019).

Targeted therapies have shifted the MG treatment paradigm toward precision medicine, offering critical alternatives for patients who are refractory to conventional immunosuppressants (Cavalcante et al. 2024). Downstream approaches include complement inhibitors that prevent terminal cascade damage in patients with AChR-positive MG and FcRn antagonists that accelerate the clearance of pathogenic IgG (Gable and Guptill 2019; Mantegazza et al. 2020).

Conversely, upstream B-cell depletion is highly effective in individuals with MuSK-MG, although its success in AChR-MG patients may be limited by the presence of long-lived plasma cells (Yang et al. 2024; Díaz-Manera et al. 2012; Hill et al. 2008).

### Possible viral involvement

Viral infections have been implicated in the pathogenesis of several neuromuscular disorders through complex interactions involving viral pathogens, the immune system, and neuromuscular tissues. Among the viruses most frequently associated with these conditions are enteroviruses, flaviviruses, herpesviruses, and, more recently, SARS-CoV-2 (Singh et al. 2025). Neuromuscular involvement may result either from the direct viral invasion of motor neurons or muscle fibers, as reported for poliovirus and SARS-CoV-2, or, more commonly, from immune-mediated mechanisms driven by dysregulated immune responses and persistent inflammation. Indeed, accumulating evidence suggests that immune-mediated processes, rather than direct viral cytopathic effects, account for the majority of virus-associated neuromuscular disorders (Singh et al. 2025). In this context, virus-induced immune dysregulation may contribute to both the initiation and persistence of autoimmunity in patients with MG by promoting chronic inflammation, impairing immune tolerance, and facilitating the activation and expansion of autoreactive lymphocytes. Consistent with its multifactorial etiology, MG is thought to arise from a complex interplay between genetic predisposition and environmental triggers. Among the latter, infectious agents, particularly viruses, have been proposed as potential contributors to intrathymic immune dysregulation in patients with AChR-MG, possibly through the aberrant activation of TLR signaling pathways (Cavalcante et al. 2010a, b, 2016, 2017, 2018). Viral infections may therefore promote the onset and progression of autoimmune responses through multiple, nonmutually exclusive mechanisms, including the induction of local thymic inflammation, activation of innate immune pathways, molecular mimicry, and bystander activation (Johnson and Jiang 2023).

These mechanisms appear to be particularly relevant for viruses capable of persisting within immune-privileged or lymphoid tissues, such as Epstein–Barr virus (EBV), other human herpesviruses, and parvovirus B19. More specifically, the chronic thymic inflammation observed in most MG patients suggests that persistent viruses may contribute to intrathymic etiologic mechanisms of the disease (Cavalcante et al. 2011). In this context, studies have focused primarily on the thymus and gut. The thymus is a primary lymphoid organ essential for immune system maturation, particularly T-cell development and the elimination of autoreactive T-cell clones. After viral infection, the thymic microenvironment may fail to effectively eliminate autoreactive T cells, thereby facilitating autoimmunity. Persistent DNA viruses with thymic tropism, such as EBV and parvovirus B19, have been detected within thymic epithelial cells, B cells, and ectopic germinal centers, supporting their potential role in sustaining local immune activation. Moreover, EBV and cytomegalovirus (CMV) have been suggested to activate innate immune signaling within the thymus, generating a proinflammatory milieu that favors the loss of self-tolerance and promotes autoimmunity. Beyond the thymus, the gut has emerged as an additional key site involved in MG pathophysiology. Viral infection may contribute to gut dysbiosis, and MG patients often exhibit an altered gut microbiome characterized by reduced bacterial diversity, the depletion of specific taxa (e.g., Firmicutes) and the enrichment of phyla involved in immune modulation and inflammation (e.g., Proteobacteria and Bacteroidetes) (Qiu et al. 2018). This altered microbial landscape is associated with systemic inflammation and may challenge the immune balance, thereby promoting or exacerbating autoimmune mechanisms in patients with MG (Deng et al. 2025).

Molecular mimicry may occur when viral antigens share structural similarity with NMJ proteins (e.g., AChR), as hypothesized for several herpesviruses (e.g., varicella zoster virus (VZV) and herpes simplex virus-1 (HSV1)) and RNA viruses (e.g., West Nile virus (WNV) and dengue virus (DENV)), whereas intense inflammation induced by antiviral responses can initiate or potentiate autoimmune processes, ultimately disrupting immunological self-tolerance.

**Fig. 1:**
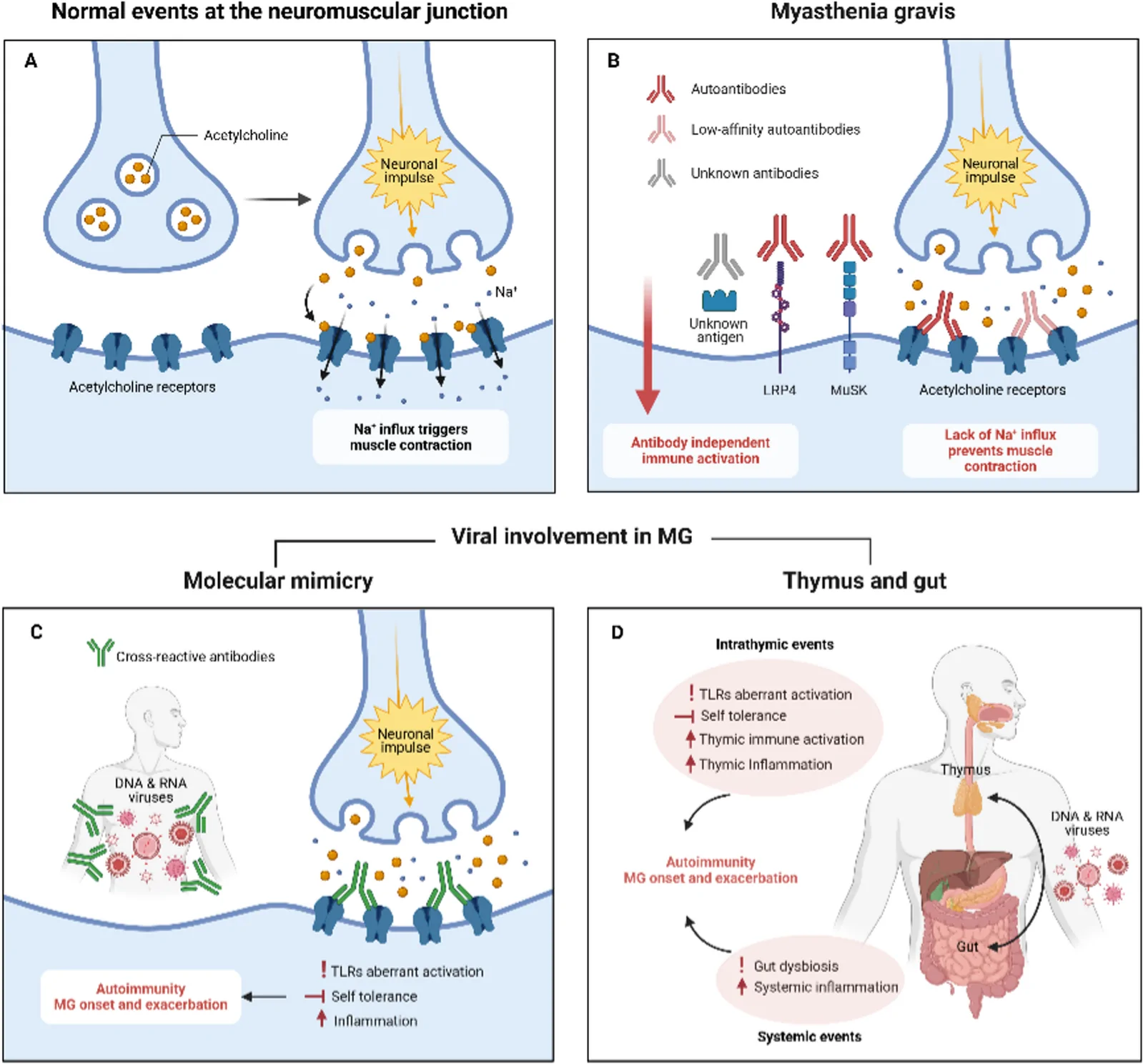
Viral involvement in myasthenia gravis. Normal events at the neuromuscular junction (Panel **A**); the impairment of synaptic transmission at the neuromuscular junction (NMJ) typical of myasthenia gravis (MG) (Panel **B**); and viral molecular mimicry as a mechanism related to MG pathogenesis are shown. The image shows only cross-reactive antibodies directed against the acetylcholine receptor (AChR) for illustrative purposes (Panel **C**); thymus and gut viral infections are associated with intrathymic and systemic events related to MG pathogenesis (Panel **D**). Images were adapted from the original images created by Ruslan Medzhitov (Creator), Akiko Iwasaki and Sally Kim (created in https://BioRender.com) (reference: Janeway’s Immunology, 8ed. © Garland Science 2012)

TLRs play a central role in the molecular pathways involved in the latter scenario. These innate immune sensors detect conserved pathogen-associated patterns and initiate downstream signaling cascades that shape the inflammatory response (Li et al. 2021). Altered TLR expression has been reported in the peripheral blood mononuclear cells and thymic tissues of MG patients, with TLR9 levels correlating with disease severity (Wang et al. 2013). Moreover, both TLR7 and TLR9 are overexpressed in the thymus of patients with MG, especially in the presence of active EBV infection (Cavalcante et al. 2016), where they may promote aberrant B-cell activation and sustain intrathymic autoimmunity (Cavalcante et al. 2018). Similar pathways have been implicated in the response to other viral nucleic acids (Chen et al. 2024), supporting the hypothesis of a convergent inflammatory mechanism rather than distinct virus-specific pathways and providing a common framework for several of the viral associations discussed below (Fig. 1).

### Myasthenia gravis and DNA viruses

Overall, the data from the literature, which is described in detail in the following paragraphs, highlight pathogenic links between persistent viral infections (EBV and poliovirus), chronic inflammation, B-cell dysregulation with GC formation in the thymus of patients with MG, and the development of autoimmunity (Cavalcante et al. 2013). Specifically, the chronic intrathymic inflammation characteristic of follicular hyperplasia that is potentially triggered or sustained by viral infection may promote the infiltration of B and T lymphocytes into the thymus, facilitate bystander activation, and induce aberrant immune cell activation. These processes can ultimately lead to autosensitization against locally expressed autoantigens, such as acetylcholine receptors (AChRs), which are expressed by thymic epithelial cells (TECs) and myoid cells.

Furthermore, a disruption of B-cell tolerance resulting from chronic inflammation and viral reactivation, particularly EBV reactivation, has been proposed as a key mechanism driving the abnormal activation and expansion of B cells, including autoreactive clones that produce autoantibodies. This process contributes to the maintenance of autoimmune responses within the thymus and supports their persistence in the peripheral immune system (Cavalcante et al. 2013).

### Myasthenia gravis and human herpesviruses

#### Epstein–Barr virus

Among all known viruses, EBV has been proposed as a primary candidate that induces autoimmune disorders, including MG, because of its ability to promote abnormal B-lymphocyte activation and survival (Niller et al. 2011; Cavalcante et al. 2016). Specifically, four distinct forms of latency have been described, each characterized by a unique expression pattern of viral proteins and EBV-encoded RNAs (EBERs) (Cavalcante et al. 2016; Robinson et al. 2024). Several studies have reported the presence of EBV in preoperative analyses of thymoma patients, indicating an association between the virus and thymic malignancies (Zhao et al. 2023; He et al. 2025).

Notably, one study revealed a significantly higher frequency of EBV detection in patients with MG-associated thymomas than in those with non-MG thymomas, with EBV DNA and EBER1 positivity rates reaching 53.8% and 84.6%, respectively (Cavalcante et al. 2017). Most EBV-positive thymomas are characterized by a previous latent infection, which is supported by the isolation of EBV latency proteins and the concomitant absence of latent EBNA2, late gp350/220, and early BZLF1 lytic transcripts (Cavalcante et al. 2017).

Furthermore, the presence of EBV-infected B cells within the thymic tissue of MG patients has been frequently described. McGuire et al. (1988) detected EBV in 4 of 6 (66.7%) MG patients and 2 of 5 (40%) non-MG patients. In 2010, Cavalcante et al. reported the abnormal accumulation of B cells and plasma cells harboring EBV in thymuses from patients with MG; specifically, they isolated viral DNA and both lytic and latent transcripts and proteins from both hyperplastic and involuted thymuses from MG patients. These factors were absent in control thymuses, supporting an association between EBV infection and chronic B-cell activation in the thymus of patients with MG, although a causal relationship remains unproven (Cavalcante et al. 2010a, b). Similarly, a subsequent study by the same group revealed EBV DNA and latent/lytic transcripts in 12/19 (63%) thymuses from patients with MG (Cavalcante et al. 2011). Moreover, a significant association between high titers of anti-EBV nuclear antigen 1 (EBNA-1) antibodies and early-onset MG has been reported (Csuka et al. 2012).

However, some studies failed to support an association between EBV and the initiation of MG (Meyer et al. 2011; Kakalacheva et al. 2011; Jing et al. 2013). For instance, Meyer et al. (2011) reported no evidence of lytic or latent EBV infection in 25 patients with early-onset MG, nor was evidence of EBV infection found in thymic samples from Chinese patients with MG (Jing et al. 2013). Additionally, another study revealed only minimal levels of EBV DNA in 6/16 (37.5%) thymic specimens from MG patients, suggesting that viral replication is rare in individuals with MG (Kakalacheva et al. 2011). Several limitations may explain these conflicting findings. Tissue-based studies reporting a positive association were conducted on small cohorts, thereby limiting their statistical power and reliability (McGuire et al. 1988; Cavalcante et al. 2010a, b, 2011, 2017). Furthermore, the use of different detection strategies, including PCR-based assays, EBER1 in situ hybridization, and immunohistochemistry for lytic and latent proteins, hampers direct comparisons across studies. Although variations in assay sensitivity could contribute to these discrepancies, Kakalacheva et al. (2011) failed to support a role for EBV in the initiation of MG despite the use of a highly sensitive PCR assay. Conversely, concerns have been raised regarding the specificity of positive findings, particularly the nuclear staining observed with anti-LMP1 antibodies, which is a recognized immunohistochemical artifact (Jing et al. 2013). Tissue selection represents another potential source of variability, since most studies reporting a positive association analyzed fresh or snap-frozen thymic tissue, whereas studies reporting a negative association relied on formalin-fixed paraffin-embedded (FFPE) specimens. Notably, studies reporting a negative association verified both nucleic acid integrity and proper assay performance. Furthermore, compared with fresh frozen tissue, FFPE material is more prone to contamination, thereby increasing the risk of false positives and reinforcing the reliability of negative data (Jing et al. 2013). Finally, given the high seroprevalence of EBV in the adult population, the detection of viral DNA or elevated anti-EBV antibody titers may reflect latent bystander infection in infiltrating B lymphocytes rather than active viral involvement in disease pathogenesis. Overall, these considerations underscore the need for larger studies employing standardized tissue sampling and viral detection methods to clarify the role of EBV in MG. Despite these limitations, several biologically plausible mechanisms have been proposed to explain how EBV could contribute to MG pathogenesis, including the modulation of TLR-mediated immune responses, the generation of autoreactive T-cell clones during infectious mononucleosis, antigenic cross-reactivity between viral and self-antigens, and the immortalization of preexisting autoreactive B cells (Niller et al. 2011). Furthermore, type III latency stimulates the production of inflammatory cytokines and potent T-cell activation, with infected cells acting as antigen-presenting cells (APCs) that drive the proliferation of autoreactive B and T cells (Morawiec et al. 2025).

#### Other human herpesviruses

In terms of human alpha herpesviruses, some studies have reported that VZV infection rarely occurs before the onset of MG, particularly in children. Different case reports have described the onset of acute ocular or oculobulbar weakness approximately two weeks after a primary varicella infection, with clinical features including fluctuating ptosis, fatigable extraocular weakness, and a characteristic decrease in low-frequency repetitive nerve stimulation, despite the absence of detectable anti-AChR antibodies (Saha et al. 2007). Additional observations from pediatric patients, including cases of MG developing shortly after varicella infection, suggest that VZV-induced immune activation may contribute to disease initiation (Felice et al. 2005). Evidence from broader viral-associated MG cohorts has also revealed that herpes zoster infection precedes MG onset in adults, suggesting that both primary VZV infection and reactivation may trigger neuromuscular autoimmunity in susceptible individuals (Korn and Abramsky 1981).

Furthermore, a mechanistic model of the involvement of HSV-1/2 in MG pathogenesis was proposed. On the basis of a molecular mimicry hypothesis, autoantibodies from MG patients have been shown to bind an HSV1 glycoprotein D epitope homologous to a region of the AChR alpha subunit, suggesting a potential cross-reactive autoimmune trigger (Korn and Abramsky 1981). However, epidemiological studies have not reported increased HSV1/2 prevalence in patients with MG, and the available clinical data do not support HSV as a common initiating factor.

The first scientific report on beta herpesviruses appeared in 1978 and suggested that persistent thymic infection, most notably by CMV, might induce a loss of tolerance through antigenic alteration of thymic epithelial cells and subsequent cross‑reactive autoantibody production (Tindall et al. 1978). These findings contrast with those of a serological analysis, which did not reveal significant differences in CMV antibody titers between a cohort of 104 MG patients and matched controls (Klavinskis et al. 1985). More recently, a serological study conducted by Probst et al. showed that compared with healthy controls, MG patients exhibit significantly elevated CMV‑specific IgG titers (up to eightfold higher) and stronger reactivity to CMV nuclear antigens and pp52, suggesting possible chronic or recurrent CMV stimulation of the immune system (Victoria Probst 2021). Finally, the case reported by Mori et al. illustrates a rare but clinically informative example of dual autoimmune neuromuscular involvement, in which a patient developed both chronic inflammatory demyelinating polyneuropathy (CIDP) and MG shortly after a CMV infection. Elevated CMV IgM/IgG titers, in addition to increasing anti-AChR antibody levels, suggest potential postinfectious autoimmune activation (Mori et al. 2006).

Other herpesviruses, such as human herpesvirus 6 (HHV6) and human herpesvirus 7 (HHV7), are frequently detected in thymic tissue from both MG patients and non-MG controls. Recent analyses of fresh thymic samples revealed that HHV6 and HHV7 DNA were present at similar frequencies across MG and control tissues but were not enriched in patients with MG, indicating that their thymic persistence likely reflects background viral latency rather than a pathogenic trigger (Nowlan et al. 2025). Although HHV6 has the capacity to deplete thymocytes in experimental models, raising theoretical concerns about its impact on thymic immune regulation, similar effects have not been substantiated in humans with MG (Nowlan et al. 2025).

#### Other DNA viruses

Parvovirus B19 has shown the most consistent association with MG, as multiple studies have demonstrated the presence of parvovirus B19 DNA and VP1/VP2 proteins within thymic epithelial cells and ectopic germinal centers in thymic hyperplasia and thymoma, supporting a possible association with B-cell hyperplasia and intrathymic autoimmune activation (Gong et al. 2019, 2023). Emerging evidence has also indicated that human polyomaviruses (HPyVs), especially HPyV7, are detected in 54–62% of both patients with MG‑positive and MG‑negative thymoma, with viral protein expression localized primarily to neoplastic thymic epithelial cells (Rennspiess et al. 2015). Merkel cell polyomavirus DNA was detected in 7 of 36 (19.4%) individuals with thymomas, 3 of which showed weak expression at the viral protein level (Chteinberg et al. 2019).

### Myasthenia gravis and RNA viruses

#### SARS-CoV-2

Among the human coronaviruses hypothesized to contribute to autoantibody production in diverse autoimmune diseases, SARS-CoV-2 has attracted increasing attention because of the numerous reports of patients diagnosed with coronavirus disease 2019 (COVID-19) who subsequently developed multiple types of autoantibodies and autoimmune conditions. In patients with no prior history, the virus can induce new-onset MG, which typically appears 2 to 6 weeks after the initial infection. In patients with established MG, COVID-19 often acts as a precipitating factor for exacerbations or myasthenic crises, regardless of whether the patient is seropositive or seronegative for anti-AChR/anti-MuSK antibodies. In the first group of studies, SARS-CoV-2 infection was hypothesized to be a potential trigger of previously undiagnosed MG, both the generalized form and the ocular form. Cases have been identified in patients who are seropositive for anti-AChR antibodies or anti-titin antibodies or who are seronegative. In the second group of studies, SARS-CoV-2 infection was recognized as a precipitating factor for the exacerbation of MG symptoms in myasthenic patients independent of their serological status. Case reports corresponding to both groups of studies are summarized in Table 1.

Several broader studies on SARS-CoV-2-induced MG exacerbation have also been published. Briefly, in 2020, an effort to monitor the clinical course and outcomes of MG in patients with laboratory-confirmed or clinically suspected COVID-19 led to the development of the physician-reported CARE-MG registry (COVID-19 Associated Risks and Effects in Myasthenia Gravis) by the International MG/COVID-19 Working Group and collaborating neurologists. As of October 2020, 36 of the 91 MG patients included in the registry had developed either a myasthenic crisis or MG exacerbation, requiring rescue therapy (Muppidi et al. 2020). In another retrospective study, a population of 39 MG patients admitted to Emory University (Atlanta, Georgia, USA) who tested positive for SARS-CoV-2 infection was analyzed by Thomas et al. (Thomas et al. 2023). Five of the 39 patients experienced an MG exacerbation, defined as worsening of the Myasthenia Gravis Foundation of America (MGFA) score, with typical symptoms such as diplopia, ptosis, dysphagia, dysarthria or weakness. Six of the eight patients experienced an MG exacerbation, as evidenced by an increase in the Myasthenia Gravis Foundation of America (MGFA) score, indicating clinical deterioration from previously stable disease. Further monocenter observational retrospective studies reported an MG exacerbation following SARS-CoV-2 infection in a total of seven patients with a known history of MG who were hospitalized for COVID-19 (Saied et al. 2021).

Notably, several patients with MG who experienced no consequences for their myasthenic clinical course during or after SARS-CoV-2 infection have been documented (Aksoy and Oztutgan 2020; Ramaswamy and Govindarajan 2020).

**Table 1:** Published case reports on SARS-CoV-2 and new onset/exacerbation of MG

Study Author/Year	New-Onset or Existing MG	Antibody Status	Timing of MG Symptoms
(Restivo et al. 2020)	New-Onset	Anti-AChR (+)	5–7 days after fever onset from COVID-19
(Chatterjee et al. 2022)	New-Onset	Anti-AChR (+)	1 month after hospital admission for COVID-19
(Muralidhar Reddy et al. 2021)	New-Onset	Anti-AChR (+)	6 weeks after a positive RT‒PCR test for SARS-CoV-2
(Karimi et al. 2021)	New-Onset	Anti-AChR (+)	4 to 6 weeks after hospital admission for COVID-19
(Tereshko et al. 2023)	New-Onset	Anti-AChR (+)	13 days after a positive RT‒PCR test for SARS-CoV-2
(Mincă et al. 2024)	New-Onset (Unmasked)	Anti-AChR (+)	5 days after a COVID-19 diagnosis
(Huber et al. 2020)	New-Onset (Ocular)	Anti-AChR (+)	3 weeks after COVID-19 symptoms
(Sriwastava et al. 2021)	New-Onset (Ocular)	Anti-AChR (+)	Concurrent with active SARS-CoV-2 infection
(Jõgi et al. 2022)	New-Onset	AChR (+)/Titin (+)	14 days after COVID-19 symptoms appeared
(Castro Silva et al. 2024)	New-Onset	Anti-AChR (+)/Anti-Titin (+)/Anti-MuSK (+)	2 weeks after mild respiratory symptoms appeared
(Delly et al. 2020)	Existing (Exacerbation)	Anti-AChR (+)	Concurrent with COVID-19 symptoms
(Moschella and Roth 2020)	Existing (Exacerbation)	Anti-AChR (+)	3 days after admission for MG symptoms
(Singh and Govindarajan 2020)	Existing (Exacerbation)	Anti-AChR (-)/Anti-Titin (-)	Concurrent with active SARS-CoV-2 infection

#### Flaviviruses

The possible correlation between flaviviruses and MG infection has been described in previous case reports. DENV serotypes 2 and 3 are linked to neurological complications, such as MG, during the febrile stage of infection (Verma et al. 2014). Li et al. investigated the risk of developing autoimmune diseases in patients with DENV infection in a cohort study of 12,506 newly diagnosed DENV-positive patients and 112,554 controls who were enrolled between 2000 and 2010 and followed for 3 years. Compared with control patients, patients infected with DENV had a higher risk of developing autoimmune diseases, such as MG (Li et al. 2018). At least two case reports described a correlation between DENV infection and MG development: one published by Nata Pramata et al. (Nata Pratama et al. 2014) and the other by Lau and Chinnasami (Lau and Chinnasami 2021). In both cases, the patients developed neurological symptoms, and laboratory tests confirmed DENV infection and the presence of anti-AChR antibodies, a key marker of MG, in the serum.

WNV infection was also investigated with respect to its association with MG. A retrospective study was conducted on 107 patients with a confirmed neuroinvasive form of WNV infection who had never shown MG symptoms prior to viral infection (Leis et al. 2014). Six patients developed MG symptoms 3 to 7 months after infection. The authors suggested that the potential cross-reactivity of human antibodies against WNV with AChR subunits can mediate MG onset. In the other 29 MG patients with high levels of antibodies against AChR, the presence of anti-WNV antibodies (Greco et al. 2016) was higher than that in the control group. Interestingly, an association only appears to exist between neuroinvasive WNV infection and MG onset (Altay et al. 2017). Like DENV, WNV infection may also be involved in the exacerbation of MG symptoms (Hawkes et al. 2018). Zika virus, another member of the *Flaviviridae* family, is usually associated with mild symptoms, even though it has been correlated with neurological disorders (Molko et al. 2017). Molko et al. described two patients with a confirmed ZKV infection who developed thymoma with high levels of anti-AChR antibodies (Molko et al. 2017).

#### Other RNA viruses

Subsequent studies have reported possible correlations between MG and other RNA viruses. Among *Picornaviruses*, Cavalcante et al. reported the detection of enteroviral RNA and the viral capsid protein VP1 in the thymic tissue of a subset of MG patients, particularly those with thymitis or thymoma, whereas none of the thymus samples from control subjects tested positive. Moreover, the detection of both plus and minus viral RNA strands in the positive samples suggests persistent infection. These findings are consistent with those of previous reports describing the presence of persistent enteroviral RNA in patients with neuropathic and muscular disorders (Cavalcante et al. 2010a, b). With respect to *Retroviruses*, several case reports have described anti-MuSK MG onset following immune reconstitution in HIV-infected individuals receiving antiretroviral therapy (Carmillo et al. 2023; Ragunathan et al. 2015; Nair 2025; Heckmann and Marais 2020), whereas HTLV-I, a neurotropic retrovirus associated with myelopathy/tropical spastic paraparesis (HAM/TSP), has been reported concomitantly with MG and detected at high frequency in the thymic tissue of MG patients (Levin et al. 2002; Lalive et al. 2007). Among *hepatitis viruses*, the strongest evidence has been obtained for hepatitis E virus (HEV), with a higher incidence of thymoma in newly diagnosed MG patients who are HEV positive (Wang et al. 2018). Temporal associations with MG have also been described for acute HEV and HAV infections, whereas no significant correlation has been observed for hepatitis C virus (HCV) (Belbezier et al. 2014; Ks et al. 2023; Halfon et al. 1996). In the case of *Togaviruses*,* s*evere neurological manifestations of Chikungunya virus (CHIKV) infection have been reported in a patient with pre-existing MG and immunosuppression (Uruçu et al. 2022). Finally, among *Orthomyxoviruses*, one study described an increased severity of MG symptoms in patients with influenza A infection compared with those with the common cold (Seok et al. 2017).

### Conclusions and future perspectives

Considerable evidence supports a link between infection-driven immune activation and autoimmunity in patients with MG, including the following: (i) the thymus is particularly susceptible to infections (Savino 2006); (ii) infections are well recognized as triggers of MG exacerbations and myasthenic crises in the clinic (Gilhus et al. 2018); (iii) experimental models (EAMGs) have shown that immune stimulation with adjuvants is critical for triggering anti-AChR autoimmunity (Losen et al. 2015); and (iv) pathogen-derived components/TLR agonists can lead to MG-like disease in mice (Losen et al. 2015; Robinet et al. 2017). To date, although several pathogens have been associated with either the development or exacerbation of MG, none have been definitively shown to cause this disease. As emphasized by Leopardi et al. (Leopardi et al. 2021), well-designed observational studies with adequate sample sizes, along with mechanistic research, are still needed to clarify the contribution of viral infections to the onset of MG and to strengthen the evidence for a causal or triggering relationship between pathogen infection and MG.

## Data Availability

No datasets were generated or analysed during the current study.

## References

[CR1] Aksoy E, Oztutgan T (2020) COVID-19 Presentation in Association with Myasthenia Gravis: A Case Report and Review of the Literature. Case Rep Infect Dis 2020:8845844 10.1155/2020/8845844 PMC7436352 32850160

[CR2] Alahgholi-Hajibehzad M, Kasapoglu P, Jafari R, Rezaei N (2015) The role of T regulatory cells in immunopathogenesis of myasthenia gravis: implications for therapeutics. Expert Rev Clin Immunol 11:859–870 10.1586/1744666X.2015.1047345 25973691

[CR3] Altay FA, Güven H, Şentürk G, Ferik S, Şencan İ, Çomoğlu S (2017) Is West Nile Virus infection associated with myasthenia gravis? Trop Doct 47:30–34 10.1177/0049475516655069 27342918

[CR4] Belbezier A, Deroux A, Sarrot-Reynauld F, Larrat S, Bouillet L (2014) Myasthenia gravis associated with acute hepatitis E infection in immunocompetent woman. Emerg Infect Dis 20:908–910 10.3201/eid2005.131551 PMC4012809 24751295

[CR5] Carmillo L, Congedo P, Caggiula M, Nuzzo MM, Fasano A (2023) MuSK myasthenia gravis as a manifestation of immune reconstitution inflammatory syndrome in an HIV-positive patient: a challenging diagnosis and therapeutic approach. Neurol Sci 44:4575–4577 10.1007/s10072-023-07002-5 37594551

[CR6] Castro Silva B, Saianda Duarte M, Rodrigues Alves N, Vicente P, Araújo J (2024) Seronegative Myasthenia Gravis: A Rare Disease Triggered by SARS-CoV-2 or a Coincidence? Cureus 16:e67511 10.7759/cureus.67511 PMC11417286 39314596

[CR7] Cavalcante P, Barberis M, Cannone M, Baggi F, Antozzi C, Maggi L, Cornelio F, Barbi M, Didò P, Berrih-Aknin S, Mantegazza R, Bernasconi P (2010a) Detection of poliovirus-infected macrophages in thymus of patients with myasthenia gravis. Neurology 74:1118–1126 10.1212/WNL.0b013e3181d7d884 20368632

[CR8] Cavalcante P, Barzago C, Baggi F, Antozzi C, Maggi L, Mantegazza R, Bernasconi P (2018) Toll-like receptors 7 and 9 in myasthenia gravis thymus: amplifiers of autoimmunity? Ann N Y Acad Sci 1413:11–24 10.1111/nyas.13534 29363775

[CR9] Cavalcante P, Cufi P, Mantegazza R, Berrih-Aknin S, Bernasconi P and R. Le Panse (2013) Etiology of myasthenia gravis: innate immunity signature in pathological thymus. Autoimmun Rev 12:863–874 10.1016/j.autrev.2013.03.010 23535157

[CR10] Cavalcante P, Galbardi B, Franzi S, Marcuzzo S, Barzago C, Bonanno S, Camera G, Maggi L, Kapetis D, Andreetta F, Biasiucci A, Motta T, Giardina C, Antozzi C, Baggi F, Mantegazza R, Bernasconi P (2016) Increased expression of Toll-like receptors 7 and 9 in myasthenia gravis thymus characterized by active Epstein-Barr virus infection. Immunobiology 221:516–527 10.1016/j.imbio.2015.12.007 26723518

[CR11] Cavalcante P, Maggi L, Colleoni L, Caldara R, Motta T, Giardina C, Antozzi C, Berrih-Aknin S, Bernasconi P, Mantegazza R (2011) Inflammation and epstein-barr virus infection are common features of myasthenia gravis thymus: possible roles in pathogenesis. Autoimmune Dis 2011:213092 10.4061/2011/213092 PMC3180177 21961056

[CR12] Cavalcante P, Mantegazza R, Antozzi C (2024) Targeting autoimmune mechanisms by precision medicine in Myasthenia Gravis. Front Immunol 15:1404191 10.3389/fimmu.2024.1404191 PMC11187261 38903526

[CR13] Cavalcante P, Marcuzzo S, Franzi S, Galbardi B, Maggi L, Motta T, Ghislandi R, Buzzi A, Spinelli L, Novellino L, Baggi F, Antozzi C, Conforti F, De Pas TM, Barberis M, Bernasconi P, Mantegazza R (2017) Epstein-Barr virus in tumor-infiltrating B cells of myasthenia gravis thymoma: an innocent bystander or an autoimmunity mediator? Oncotarget 8:95432–95449 10.18632/oncotarget.20731 PMC5707033 29221139

[CR14] Cavalcante P, Serafini B, Rosicarelli B, Maggi L, Barberis M, Antozzi C, Berrih-Aknin S, Bernasconi P, Aloisi F, Mantegazza R (2010b) Epstein-Barr virus persistence and reactivation in myasthenia gravis thymus. Ann Neurol 67:726–738 10.1002/ana.21902 20517934

[CR15] Chatterjee T, Senthil Kumaran S, Roy M (2022) A Case Report and Literature Review of New-Onset Myasthenia Gravis After COVID-19 Infection. Cureus 14:e33048 10.7759/cureus.33048 PMC9881688 36721575

[CR17] Chen Y, Zhang XS, Wang YG, Lu C, Li J, Zhang P (2021) Imbalance of Th17 and Tregs in thymoma may be a pathological mechanism of myasthenia gravis. Mol Immunol 133:67–76 10.1016/j.molimm.2021.02.011 33636431

[CR16] Chen YH, Wu KH, Wu HP (2024) Unraveling the complexities of toll-like receptors: from molecular mechanisms to clinical applications. Int J Mol Sci 25 10.3390/ijms25095037 PMC11084218 38732254

[CR18] Chteinberg E, Klufah F, Rennspiess D, Mannheims MF, Abdul-Hamid MA, Losen M, Keijzers M, De Baets MH, Kurz AK and A. Zur Hausen (2019) Low prevalence of Merkel cell polyomavirus in human epithelial thymic tumors. Thorac Cancer 10:445–451 10.1111/1759-7714.12953 PMC6397898 30628176

[CR19] Csuka D, Banati M, Rozsa C, Füst G, Illes Z (2012) High anti-EBNA-1 IgG levels are associated with early-onset myasthenia gravis. Eur J Neurol 19:842–846 10.1111/j.1468-1331.2011.03636.x 22221650

[CR20] Delly F, Syed MJ, Lisak RP, Zutshi D (2020) Myasthenic crisis in COVID-19. J Neurol Sci 414:116888 10.1016/j.jns.2020.116888 PMC7200364 32413767

[CR22] Deng X, Gong X, Zhou D (2025) Perturbations in gut microbiota composition in patients with autoimmune neurological diseases: a systematic review and meta-analysis. Front Immunol 16:1513599 10.3389/fimmu.2025.1513599 PMC11839609 39981228

[CR24] Díaz-Manera J, Martínez-Hernández E, Querol L, Klooster R, Rojas-García R, Suárez-Calvet X, Muñoz-Blanco JL, Mazia C, Straasheijm KR, Gallardo E, Juárez C, Verschuuren JJ, Illa I (2012) Long-lasting treatment effect of rituximab in MuSK myasthenia. Neurology 78:189–193 10.1212/WNL.0b013e3182407982 22218276

[CR23] Dragin N, Le Panse R (2025) Thymic physiology and pathophysiology in Myasthenia Gravis. Int Rev Neurobiol 182:67–88 10.1016/bs.irn.2025.04.023 40675741

[CR25] Felice KJ, DiMario FJ, Conway SR (2005) Postinfectious myasthenia gravis: report of two children. J Child Neurol 20:441–444 10.1177/08830738050200051501 15968930

[CR26] Gable KL, Guptill JT (2019) Antagonism of the Neonatal Fc Receptor as an Emerging Treatment for Myasthenia Gravis. Front Immunol 10:3052 10.3389/fimmu.2019.03052 PMC6965493 31998320

[CR27] Gilhus NE, Breiner A (2025) Epidemiology of myasthenia gravis. Int Rev Neurobiol 182:161–196 10.1016/bs.irn.2025.04.028 40675734

[CR28] Gilhus NE, Romi F, Hong Y, Skeie GO (2018) Myasthenia gravis and infectious disease. J Neurol 265:1251–1258 10.1007/s00415-018-8751-9 29372387

[CR29] Gong L, Li Y, Li X, Tu Q, Mou X, Wang S, Wan Y, Lu Q, Wang J, Zhang W, Zhu S, Han X, Yao L, Zhang J, Huang G (2019) Detection of human parvovirus B19 infection in the thymus of patients with thymic hyperplasia-associated myasthenia gravis. Clin Microbiol Infect 25:109e7–09e12 10.1016/j.cmi.2018.03.036 29649594

[CR30] Gong L, Tian J, Zhang Y, Feng Z, Wang Q, Wang Y, Zhang F, Zhang W, Huang G (2023) Human Parvovirus B19 May Be a Risk Factor in Myasthenia Gravis with Thymoma. Ann Surg Oncol 30:1646–1655 10.1245/s10434-022-12936-9 PMC9744379 36509875

[CR31] Gradolatto A, Nazzal D, Truffault F, Bismuth J, Fadel E, Foti M, Berrih-Aknin S (2014) Both Treg cells and Tconv cells are defective in the Myasthenia gravis thymus: roles of IL-17 and TNF-α. J Autoimmun 52:53–63 10.1016/j.jaut.2013.12.015 24405842

[CR32] Greco M, Cofano P, Lobreglio G (2016) Seropositivity for West Nile Virus Antibodies in Patients Affected by Myasthenia Gravis. J Clin Med Res 8:196–201 10.14740/jocmr2413w PMC4737029 26858791

[CR33] Halfon P, Levy M, San Marco M, Gerolami V, Khiri H, Bourliere M, Feryn JM, Gastaut JL, Pouget J, Cartouzou G (1996) Myasthenia gravis and hepatitis C virus infection. J Viral Hepat 3:329–332 10.1111/j.1365-2893.1996.tb00106.x 8947885

[CR34] Hawkes MA, Hocker SE, Leis AA (2018) West Nile virus induces a post-infectious pro-inflammatory state that explains transformation of stable ocular myasthenia gravis to myasthenic crises. J Neurol Sci 395:1–3 10.1016/j.jns.2018.09.015 30267806

[CR35] He L, Xu Y, Miao D, Zhang X (2025) Thymic Lymphoepithelial Carcinoma in Children: Report of Four Cases and Review of Literature. Fetal Pediatr Pathol 44:196–205 10.1080/15513815.2025.2486838 40242968

[CR36] Heckmann JM, Marais S (2020) Management Issues in Myasthenia Gravis Patients Living With HIV: A Case Series and Literature Review. Front Neurol 11:775 10.3389/fneur.2020.00775 PMC7472955 32973647

[CR37] Hill ME, Shiono H, Newsom-Davis J, Willcox N (2008) The myasthenia gravis thymus: a rare source of human autoantibody-secreting plasma cells for testing potential therapeutics. J Neuroimmunol 201–202:50–6 10.1016/j.jneuroim.2008.06.027 18722675

[CR38] Huber M, Rogozinski S, Puppe W, Framme C, Höglinger G, Hufendiek K, Wegner F (2020) Postinfectious Onset of Myasthenia Gravis in a COVID-19 Patient. Front Neurol 11:576153 10.3389/fneur.2020.576153 PMC7573137 33123081

[CR39] Jing F, Wei D, Wang D, Li N, Cui F, Yang F, Chen Z, Huang X (2013) Lack of Epstein-Barr virus infection in Chinese myasthenia gravis patients. Acta Neurol Scand 128:345–350 10.1111/ane.12124 23668247

[CR41] Jõgi K, Liis S, Merit R Leheste Alo-Rainer, and Vilisaar Janek (2022) New Onset Generalized Myasthenia Gravis Evolving Following SARS-CoV-2 Infection. COVID 2:464–71

[CR40] Johnson D, Jiang W (2023) Infectious diseases, autoantibodies, and autoimmunity. J Autoimmun 137:102962 10.1016/j.jaut.2022.102962 PMC10235211 36470769

[CR42] Kakalacheva K, Maurer MA, Tackenberg B, Münz C, Willcox N, Lünemann JD (2011) Intrathymic Epstein-Barr virus infection is not a prominent feature of myasthenia gravis. Ann Neurol 70:508–514 10.1002/ana.22488 21905082

[CR43] Kaminski HJ, Sikorski P, Coronel SI, Kusner LL (2024) Myasthenia gravis: the future is here. J Clin Invest 134 10.1172/JCI179742 PMC11178544 39105625

[CR44] Karimi N, Okhovat AA, Ziaadini B, Haghi Ashtiani B, Nafissi S, Fatehi F (2021) Myasthenia gravis associated with novel coronavirus 2019 infection: A report of three cases. Clin Neurol Neurosurg 208:106834 10.1016/j.clineuro.2021.106834 PMC8299214 34329810

[CR45] Khani-Habibabadi F, Roy B, Pham MC, Obaid AH, Filipek B, Nowak RJ and K. C. O’Connor (2025) AChR Autoantibody Pathogenic Properties Are Heterogeneously Distributed and Undergo Temporal Changes Among Patients With Myasthenia Gravis. Neurol Neuroimmunol Neuroinflamm 12:e200436 10.1212/NXI.0000000000200436 PMC12275905 40680247

[CR46] Klavinskis LS, Willcox N, Oxford JS, Newsom-Davis J (1985) Neurology 35:1381–1384 ‘Antivirus antibodies in myasthenia gravis’ 10.1212/wnl.35.9.1381 2991819

[CR47] Korn IL, Abramsky O (1981) Myasthenia gravis following viral infection. Eur Neurol 20:435–9U 10.1159/000115275 7308244

[CR48] Ks A, Kumar V, Ms AR, Bhat NK (2023) Acute liver failure due to hepatitis A virus presented with Guillain-Barré syndrome and ocular myasthenia gravis. BMJ Case Rep 16 10.1136/bcr-2023-254855 PMC10565331 37813556

[CR49] Lalive PH, Allali G, Truffert A (2007) Myasthenia gravis associated with HTLV-I infection and atypical brain lesions. Muscle Nerve 35:525–528 10.1002/mus.20694 17117410

[CR50] Lau YH, Chinnasami S (2021) Emergence of myasthenia gravis in dengue infection-first case report. J Neurovirol 27:183–185 10.1007/s13365-021-00950-8 33528825

[CR51] Lazaridis K, Tzartos SJ (2020) Myasthenia Gravis: Autoantibody Specificities and Their Role in MG Management. Front Neurol 11:596981 10.3389/fneur.2020.596981 PMC7734299 33329350

[CR52] Leis AA, Szatmary G, Ross MA, Stokic DS (2014) West nile virus infection and myasthenia gravis. Muscle Nerve 49:26–29 10.1002/mus.23869 23559196

[CR53] Leopardi V, Chang YM, Pham A, Luo J and O. A. Garden (2021) A Systematic Review of the Potential Implication of Infectious Agents in Myasthenia Gravis. Front Neurol 12:618021 10.3389/fneur.2021.618021 PMC8236805 34194378

[CR54] Levin MC, Lee SM, Kalume F, Morcos Y, Dohan FC, Hasty KA, Callaway JC, Zunt J, Desiderio D, Stuart JM (2002) Autoimmunity due to molecular mimicry as a cause of neurological disease. Nat Med 8:509–513 10.1038/nm0502-509 PMC2703733 11984596

[CR55] Li H, Liu S, Han J, Li S, Gao X, Wang M, Zhu J, Jin T (2021) Role of Toll-Like Receptors in Neuroimmune Diseases: Therapeutic Targets and Problems. Front Immunol 12:777606 10.3389/fimmu.2021.777606 PMC8591135 34790205

[CR56] Li HM, Huang YK, Su YC, Kao CH (2018) Increased risk of autoimmune diseases in dengue patients: A population-based cohort study. J Infect 77:212–219 10.1016/j.jinf.2018.03.014 29746944

[CR57] Losen M, Martinez-Martinez P, Molenaar PC, Lazaridis K, Tzartos S, Brenner T, Duan RS, Luo J, Lindstrom J, Kusner L (2015) Standardization of the experimental autoimmune myasthenia gravis (EAMG) model by immunization of rats with Torpedo californica acetylcholine receptors–Recommendations for methods and experimental designs. Exp Neurol 270:18–28 10.1016/j.expneurol.2015.03.010 PMC4466156 25796590

[CR58] Mantegazza R, Antozzi C (2020) From Traditional to Targeted Immunotherapy in Myasthenia Gravis: Prospects for Research. Front Neurol 11:981 10.3389/fneur.2020.00981 PMC7492201 32982957

[CR59] Mantegazza R, Cavalcante P (2019) Diagnosis and treatment of myasthenia gravis. Curr Opin Rheumatol 31:623–633 10.1097/BOR.0000000000000647 31385879

[CR60] Mantegazza R, Vanoli F, Frangiamore R, Cavalcante P (2020) Complement Inhibition for the Treatment of Myasthenia Gravis. Immunotargets Ther 9:317–331 10.2147/ITT.S261414 PMC7751298 33365280

[CR99] McGuire LJ, Huang DP, Teoh R, Arnold M, Wong K, Lee JC (1988) Epstein-Barr virus genome in thymoma and thymic lymphoid hyperplasia. Am J Pathol 131:385–390. https://pmc.ncbi.nlm.nih.gov/articles/PMC1880705/PMC1880705 2837902

[CR61] Meyer M, Höls AK, Liersch B, Leistner R, Gellert K, Schalke B, Marx A, Niedobitek G (2011) Lack of evidence for Epstein-Barr virus infection in myasthenia gravis thymus. Ann Neurol 70:515–518 10.1002/ana.22522 21905083

[CR62] Mincă A, Mincă DI, Calinoiu AL, Gheorghiță V, Popescu CC, Rusu A, Cristea AM and D. G. Mincă (2024) Myasthenia Gravis Triggered by a COVID-19 Infection: A Case Report and Literature Review. Cureus 16:e59538 10.7759/cureus.59538 PMC11144031 38827012

[CR63] Molko N, Simon O, Guyon D, Biron A, Dupont-Rouzeyrol M, Gourinat AC (2017) Zika virus infection and myasthenia gravis: report of 2 cases. Neurology 88:1097–1098 10.1212/WNL.0000000000003697 28188305

[CR64] Morawiec N, Adamczyk B, Spyra A, Herba M, Boczek S, Korbel N, Polechoński P, Adamczyk-Sowa M (2025) The role of Epstein-Barr virus in the pathogenesis of autoimmune diseases. Med (Kaunas) 61 10.3390/medicina61071148 PMC12299717 40731778

[CR65] Mori M, Kuwabara S, Nemoto Y, Tamura N, Hattori T (2006) Concomitant chronic inflammatory demyelinating polyneuropathy and myasthenia gravis following cytomegalovirus infection. J Neurol Sci 240:103–106 10.1016/j.jns.2005.08.013 16236323

[CR66] Moschella P, Roth P (2020) Isolated COVID-19 Infection Precipitates Myasthenia Gravis Crisis: A Case Report. Clin Pract Cases Emerg Med 4:524–526 10.5811/cpcem.2020.9.49049 PMC7676765 33217262

[CR67] Muppidi S, Guptill JT, Jacob S, Li Y, Farrugia ME, Guidon AC, Tavee JO, Kaminski H, Howard JF, Cutter G, Wiendl H, Maas MB, Illa I, Mantegazza R, Murai H, Utsugisawa K, Nowak RJ, CARE-MG Study Group (2020) COVID-19-associated risks and effects in myasthenia gravis (CARE-MG). Lancet Neurol 19:970–971 10.1016/S1474-4422(20)30413-0 PMC7837033 33212055

[CR68] Muralidhar Reddy Y, B SK, Osman S, Murthy JMK (2021) Temporal association between SARS-CoV-2 and new-onset myasthenia gravis: is it causal or coincidental? BMJ Case Rep 14 10.1136/bcr-2021-244146 PMC8296802 34290032

[CR69] Nair AV, Ponraj L, Sivadasan A, Aaron S (2025) When HIV meets MG: is immune reconstitution in the mix? Neuroimmunol Rep 5:100263

[CR70] Nata Pratama, Hardjo Lugito, Margaret, Andree Kurniawan, and Merlyn Tjiang (2014) Worsening muscle weakness in myasthenia gravis patient suffering dengue infection. J Trop Disease 2

[CR71] Niller HH, Wolf H, Ay E, Minarovits J (2011) Epigenetic dysregulation of epstein-barr virus latency and development of autoimmune disease. Adv Exp Med Biol 711:82–102 10.1007/978-1-4419-8216-2_7 21627044

[CR72] Nowlan K, Hannolainen L, Assimakopoulou IM, Dürnsteiner P, Sarkkinen J, Suokas S, Hedman L, Tienari PJ, Hedman K, Niku M, Aaltonen LM, Huuskonen A, Räsänen JV, Ilonen IK, Mäyränpää MI, Dunkel J, Laakso SM, Söderlund-Venermo M, Perdomo MF, Kekäläinen E (2025) Parvovirus B19 and Human Herpes Virus 6B and 7 Are Frequently Found DNA Viruses in the Human Thymus But Show No Definitive Link With Myasthenia Gravis. J Infect Dis 231:e601–e06 10.1093/infdis/jiae600 PMC11998553 39657004

[CR73] Qiu D, Xia Z, Jiao X, Deng J, Zhang L, Li J (2018) Altered Gut Microbiota in Myasthenia Gravis. Front Microbiol 9:2627 10.3389/fmicb.2018.02627 PMC6241162 30483222

[CR74] Ragunathan K, Pathak B, Dahal K (2015) MuSK myasthenia gravis as a manifestation of immune restoration disease in an HIV-positive patient. J Neurol 262:777–778 10.1007/s00415-015-7639-1 25588730

[CR75] Ramaswamy SB, Govindarajan R (2020) COVID-19 in Refractory Myasthenia Gravis- A Case Report of Successful Outcome. J Neuromuscul Dis 7:361–364 10.3233/JND-200520 PMC7369032 32508329

[CR76] Rennspiess D, Pujari S, Keijzers M, Abdul-Hamid MA, Hochstenbag M, Dingemans AM, Kurz AK, Speel EJ, Haugg A, Pastrana DV, Buck CB, De Baets MH, Zur Hausen A (2015) Detection of human polyomavirus 7 in human thymic epithelial tumors. J Thorac Oncol 10:360–366 10.1097/JTO.0000000000000390 PMC4304941 25526237

[CR77] Restivo DA, Centonze D, Alesina A, Marchese-Ragona R (2020) Myasthenia Gravis Associated With SARS-CoV-2 Infection. Ann Intern Med 173:1027–1028 10.7326/L20-0845 PMC7429993 32776781

[CR78] Robinet M, Maillard S, Cron MA, Berrih-Aknin S, Le Panse R (2017) Review on Toll-Like Receptor Activation in Myasthenia Gravis: Application to the Development of New Experimental Models. Clin Rev Allergy Immunol 52:133–147 10.1007/s12016-016-8549-4 27207173

[CR79] Robinson WH, Younis S, Love ZZ, Steinman L and T. V. Lanz (2024) Epstein-Barr virus as a potentiator of autoimmune diseases. Nat Rev Rheumatol 20:729–740 10.1038/s41584-024-01167-9 39390260

[CR80] Saha A, Batra P, Vilhekar KY, Chaturvedi P (2007) Post-varicella myasthenia gravis. Singap Med J 48:e177–e180 17538742

[CR81] Saied Z, Rachdi A, Thamlaoui S, Nabli F, Jeridi C, Baffoun N, Kaddour C, Belal S and Ben Sassi S (2021) Myasthenia gravis and COVID-19: A case series and comparison with literature. Acta Neurol Scand 144:334–40 10.1111/ane.13440 PMC8222886 33914898

[CR82] Savino W (2006) The thymus is a common target organ in infectious diseases. PLoS Pathog 2:e62 10.1371/journal.ppat.0020062 PMC1483230 16846255

[CR83] Seok HY, Shin HY, Kim JK, Kim BJ, Oh J, Suh BC, Kim SY, Kang SY, Ahn SW, Bae JS (2017) The Impacts of Influenza Infection and Vaccination on Exacerbation of Myasthenia Gravis. J Clin Neurol 13:325–330 10.3988/jcn.2017.13.4.325 PMC5653619 29057629

[CR84] Singh AK, Sudhan YG, Ramakrishna R, Durairajan SSK (2025) Viral agents in neuromuscular pathology. Int Rev Neurobiol 180:397–434 10.1016/bs.irn.2025.04.007 40414639

[CR85] Singh S, Govindarajan R (2020) COVID-19 and generalized Myasthenia Gravis exacerbation: A case report. Clin Neurol Neurosurg 196:106045 10.1016/j.clineuro.2020.106045 PMC7316067 32634699

[CR86] Sriwastava S, Tandon M, Kataria S, Daimee M, Sultan S (2021) New onset of ocular myasthenia gravis in a patient with COVID-19: a novel case report and literature review. J Neurol 268:2690–2696 10.1007/s00415-020-10263-1 PMC7549728 33047223

[CR87] Tereshko Y, Gigli GL, Pez S, De Pellegrin A, Valente M (2023) New-onset Myasthenia Gravis after SARS-CoV-2 infection: case report and literature review. J Neurol 270:601–609 10.1007/s00415-022-11472-6 PMC9645742 36352330

[CR88] Thomas EV, Bou G, Barton S, Hutto S, Garcia-Santibanez R (2023) COVID-19 infection in myasthenia gravis: Clinical course and outcomes. Muscle Nerve 68:171–175 10.1002/mus.27919 37326164

[CR89] Tindall RS, Cloud R, Luby J, Rosenberg RN (1978) Serum antibodies to cytomegalovirus in myasthenia gravis: effects of thymectomy and steroids. Neurology 28:273–277 10.1212/wnl.28.3.273 203876

[CR90] Tüzün E, Christadoss P (2013) Complement associated pathogenic mechanisms in myasthenia gravis. Autoimmun Rev 12:904–911 10.1016/j.autrev.2013.03.003 23537510

[CR91] Uruçu, Lorena M, André G, de Albuquerque C, Naurath, Marcia N, Carreira MM, Garrido VE, Emmel BE, Gama R, Binato R, Hassan, Ianick SM (2022) Chikungunya virus infection in a patient with myasthenia gravis: a case report of lethal meningoencephalitis associated with high viral load. Neuroimmunol Rep 2

[CR92] Verma R, Sahu R, Holla V (2014) Neurological manifestations of dengue infection: a review. J Neurol Sci 346:26–34 10.1016/j.jns.2014.08.044 25220113

[CR93] Victoria Probst, Nicole H, Trier GH (2021) Antibodies to cytomegalovirus are elevated in myasthenia gravis. Clin Immunol Commun 1:4–12

[CR94] Wang L, Gao F, Lin G, Yuan Y, Huang Y, Hao H, Zhuang H (2018) Association of hepatitis E virus infection and myasthenia gravis: A pilot study. J Hepatol 68:1318–1320 10.1016/j.jhep.2018.01.040 29681394

[CR95] Wang YZ, Yan M, Tian FF, Zhang JM, Liu Q, Yang H, Zhou WB, Li J (2013) Possible involvement of toll-like receptors in the pathogenesis of myasthenia gravis. Inflammation 36:121–30 10.1007/s10753-012-9526-6 22898741

[CR96] Wolfe GI, Kaminski HJ, Aban IB, Minisman G, Kuo HC, Marx A, Ströbel P, Mazia C, Oger J, Cea JG, Heckmann JM, Evoli A, Nix W, Ciafaloni E, Antonini G, Witoonpanich R, King JO, Beydoun SR, Chalk CH, Barboi AC, Amato AA, Shaibani AI, Katirji B, Lecky BRF, Buckley C, Vincent A, Dias-Tosta E, Yoshikawa H, Waddington-Cruz M, Pulley MT, Rivner MH, Kostera-Pruszczyk A, Pascuzzi RM, Jackson CE, Verschuuren JJGM, Massey JM, Kissel JT, Werneck LC, Benatar M, Barohn RJ, Tandan R, Mozaffar T, Silvestri NJ, Conwit R, Sonett JR, Jaretzki A, Newsom-Davis J, Cutter GR, MGTX Study Group (2019) Long-term effect of thymectomy plus prednisone versus prednisone alone in patients with non-thymomatous myasthenia gravis: 2-year extension of the MGTX randomised trial. Lancet Neurol 18:259–268 10.1016/S1474-4422(18)30392-2 PMC6774753 30692052

[CR97] Yang X, Zhang W, Guo J, Ma C, Li B (2024) Efficacy and safety of low-dose rituximab in the treatment of myasthenia gravis: a systemic review and meta-analysis. Front Neurol 15:1439899 10.3389/fneur.2024.1439899 PMC11461331 39385818

[CR98] Zhao L, Ding JY, Tao YL, Zhu K, Chen G (2023) Detection of Epstein-Barr virus infection in thymic epithelial tumors by nested PCR and Epstein-Barr-encoded RNA ISH. Infect Agent Cancer 18:37 10.1186/s13027-023-00497-9 PMC10257281 37296417

